# The role of the glucose-sensing transcription factor carbohydrate-responsive element-binding protein pathway in termite queen fertility

**DOI:** 10.1098/rsob.160080

**Published:** 2016-05-18

**Authors:** David Sillam-Dussès, Robert Hanus, Michael Poulsen, Virginie Roy, Maryline Favier, Mireille Vasseur-Cognet

**Affiliations:** 1 Laboratoire d'Ethologie Expérimentale et Comparée, Université Paris 13, EA4443, 93430 Villetaneuse, France; 2 UMR IRD 242, UPEC, CNRS 7618, UPMC 113, INRA 1392, PARIS 7 113, Institut d'Ecologie et des Sciences de l'Environnement de Paris, 93140 Bondy, France; 3 Sorbonne Paris Cité, Paris, France; 4 Sorbonne Universités, Paris, France; 5 Institute of Organic Chemistry and Biochemistry, Academy of Sciences of the Czech Republic, 16610 Prague, Czech Republic; 6 Centre for Social Evolution, Section for Ecology and Evolution, Department of Biology, University of Copenhagen, 2100 Copenhagen East, Denmark; 7 Institut National de la Santé et de la Recherche Médicale, Unité 1016, Institut Cochin, 75014 Paris, France

**Keywords:** reproduction, phenotypic plasticity, carbohydrate-responsive element-binding protein, transcription factor, social insects, lipogenesis

## Abstract

Termites are among the few animals that themselves can digest the most abundant organic polymer, cellulose, into glucose. In mice and *Drosophila*, glucose can activate genes via the transcription factor carbohydrate-responsive element-binding protein (ChREBP) to induce glucose utilization and de novo lipogenesis. Here, we identify a termite orthologue of ChREBP and its downstream lipogenic targets, including acetyl-CoA carboxylase and fatty acid synthase. We show that all of these genes, including ChREBP, are upregulated in mature queens compared with kings, sterile workers and soldiers in eight different termite species. ChREBP is expressed in several tissues, including ovaries and fat bodies, and increases in expression in totipotent workers during their differentiation into neotenic mature queens. We further show that ChREBP is regulated by a carbohydrate diet in termite queens. Suppression of the lipogenic pathway by a pharmacological agent in queens elicits the same behavioural alterations in sterile workers as observed in queenless colonies, supporting that the ChREBP pathway partakes in the biosynthesis of semiochemicals that convey the signal of the presence of a fertile queen. Our results highlight ChREBP as a likely key factor for the regulation and signalling of queen fertility.

## 1. Introduction

Glucose is the most widely used sugar in animals, serving in energy production and in the provision of macromolecular precursors, and glucose is a signalling molecule in the liver and fat tissues [1]. The basic helix–loop–helix transcription factor paralogues, ChREBP (carbohydrate-responsive element-binding protein, also called Mondo B) and Mondo A, respond to glucose signalling in mammals [2–8]. For glucose-induced transcriptional responses, ChREBP and Mondo A bind to the carbohydrate-response elements at the promoter regions of glycolytic and lipogenic genes, including liver pyruvate kinase (L-PK), fatty-acid synthase (FAS) and acetyl-CoA carboxylase (ACC) [2,5,7,9]. In mammals, ChREBP and Mondo A play tissue-specific roles: ChREBP functions in the liver, adipose tissue, intestines and pancreatic beta cells [10–14], while Mondo A is predominantly expressed in the skeletal muscle [15]. ChREBP-deficient mice have impaired activation of glucose-induced target genes as well as a number of dysregulated metabolic phenotypes, including elevated plasma glucose and liver glycogen levels and reduced adiposity [14]. Moreover, these mice survive poorly on high-sugar diets [14]. The *Drosophila* genome encodes only one orthologue for ChREBP/Mondo A (Mondo), and disruption of its function severely affects energy metabolism, rendering the fruit fly highly intolerant to sugars and causing lethality in the late pupal stage [16]. Because of the essential role of this transcription factor in the *Drosophila* fat body [16,17], the counterpart of mammalian liver and adipose tissue, it has been proposed that the liver and adipose tissue-specific ChREBP, rather than the muscle-specific Mondo A, represents the ancestral function of the protein [16].

**Figure 1.**
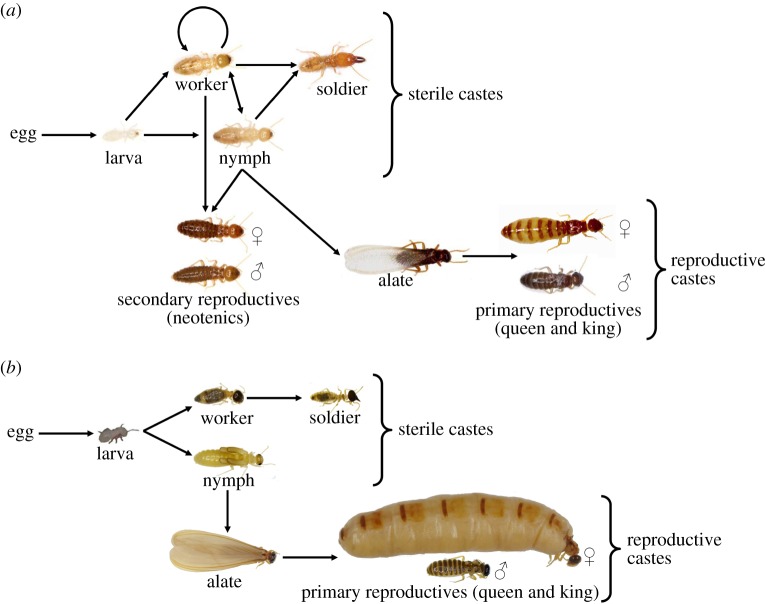
Simplified developmental pathway of (*a*) *Prorhinotermes canalifrons* and (*b*) *Nasutitermes* sp*.* with castes and life stages.

An important fact that has not been taken into consideration for the study of ChREBP so far is that glucose is the basic component of cellulose. Although cellulose is the most abundant organic polymer on Earth, only few animals can digest it. Among them are termites (Insecta: Blattodea: Termitidae), whose ecological success stems partially from their adaptation to use recalcitrant plant lignocellulose as their primary food source through symbiotic associations. Termites consume an estimated 3–7 billion tonnes of lignocellulose annually, with 74–99% of the ingested cellulose being hydrolysed [18]. Another factor contributing to the ecological success of termites is the reproductive division of labour between castes, a defining feature of all eusocial insects [19,20]. Typically, once a year, winged imagoes (also called alates) undergo a dispersal flight to become the primary reproductives (primary kings and queens) of newly founded colonies, which in some species grow to contain millions of individuals. Soldiers are permanently sterile and defend the colony. Workers participate in cooperative tasks, such as nest building, rearing of larvae, food collection and feeding of dependent nest-mates. They transfer the food to nest-mates by oral trophallaxis, and the diet transferred differs among sterile and reproductive individuals [21,22]. Many termite species, such as *Prorhinotermes canalifrons* (Rhinotermitidae; figure 1*a*), have an alternative reproductive strategy involving secondary kings and queens (also called neotenics). This reproductive option is open to immature stages and castes, such as workers (*stricto sensu* pseudergates) and nymphs, and is accompanied by the rapid development of reproductive organs and onset of reproductive activities following the moult of the immature termite.

In the honeybee, a specific diet and, more generally, the nutritional status influence caste determination and behavioural development [23,24]. The role of environment in caste determination of termites has been assumed to be omnipotent compared with exclusively genetic effects (but see [25,26]). Secondary reproductives develop in response to external stimuli, such as environmental factors (e.g. nutrition or season [20,27]) and social contexts (pheromone exposure [20]). In *Nasutitermes* sp. (Termitidae; figure 1*b*), the reproductive strategy is usually shifted towards the production of a high number of winged dispersers by a highly fecund long-lived physogastric (hypertrophied abdomen) primary queen and her mate (the primary king) assisted by numerous permanent workers and soldiers. This sophisticated colony organization, characterized by large, stable, well-defended and provisioned nests, is primarily maintained through pheromone communication that plays a major role in maintaining reproductive monopoly by the queen. In three eusocial insects (wasp, bumblebee and desert ant), a conserved class of fatty-acid derived pheromones (saturated hydrocarbons), overproduced by queens or fertile individuals, functions as sterility-inducing pheromones and as a signal of fecundity [28]. This reproductive inhibition prevents workers from activating their ovaries and causes secondary oocyte reabsorption (regression) [28]. In the termite *Cryptotermes secundus* (Kalotermitidae), queen-specific pheromones act as a fertility signal to indicate queen presence and to prevent nest-mates from reproducing [29].

Because termites use cellulose as their main carbohydrate source, we hypothesized that this dietary specialization may have placed a selective pressure on the ChREBP pathway in termites. This prompted us to investigate the role of ChREBP as a putative control factor involved in nutritionally driven regulation of the phenotypic plasticity of termite caste systems. We report here the caste-dependent expression pattern of ChREBP and its lipogenic target genes in eight different termite species. We also describe ChREBP expression during caste differentiation. Moreover, we show the impact of the diet on ChREBP gene expression, and finally, via a combination of a pharmacological approach and behavioural assays, we identify ChREBP as a fertility-signalling factor in termites.

## 2. Results

### 2.1. Carbohydrate-responsive element-binding protein is a conserved lipogenic regulator upregulated in termite queens

We performed a BLAST search using the mammalian ChREBP coding sequences as bait, and this identified a single homologous gene in the two available published termite genomes: *Zootermopsis nevadensis* [30] and *Macrotermes natalensis* [31]. To evaluate cross-species comparison, amino acid sequences of ChREBP polypeptides from *Homo sapiens*, *Mus musculus*, *Drosophila melanogaster* and *Apis mellifera* were aligned and compared with those of the termite sequences. The comparison indicated an evolutionarily-conserved termite ChREBP of 1111 amino acids with a molecular weight estimated to be 112 kDa (electronic supplementary material, figure S1). The structural organization of regulatory and functional domains, such as the glucose-sensing module (GSM), was evolutionarily conserved among all ChREBP orthologues [32]. Mammalian (*H. sapiens* and *M. musculus*) and termite (*Z. nevadensis* and *M. natalensis*) GSM ChREBP amino acid sequences exhibited 45–46% sequence identity.

**Figure 2.**
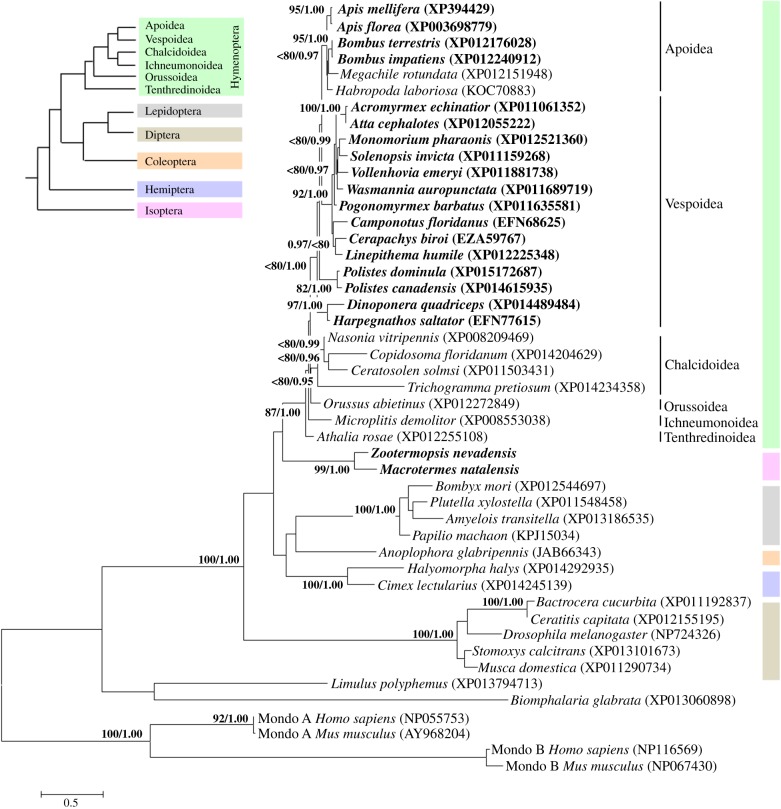
Fifty per cent majority rule consensus tree obtained from the ML analyses of ChREBP amino acid sequences (GSM region, 331 amino acids). ML bootstrap values > 80% and Bayesian posterior probabilities > 0.95 are plotted on the nodes. Tree is rooted on vertebrate Mondo A and Mondo B (ChREBP) sequences. Social species are indicated in bold. Upper-left corner: insect phylogeny adapted from Mao *et al.* [33] and Misof *et al.* [34].

Using Bayesian inference (BI) and maximum-likelihood (ML) methods of tree reconstruction, we performed a phylogenetic analysis of the GSM amino acid sequences. The alignment contained 331 amino acids from 45 sequences from vertebrates (human and mouse), arthropods including a xiphosuran, eusocial insects (bees, wasps, ants and termites) and solitary insects (wasps, sawflies, beetles, bugs, flies, moths and butterflies), and a mollusc. The two methods of phylogenetic reconstruction produced ChREBP trees with very similar topologies (figure 2 gives the ML tree; see the electronic supplementary material, figure S2 for the BI tree). The position of the termites was surprising, because this clade appeared as the sister group of the Hymenoptera (ML tree, ML bootstrap values < 80%), and part of a clade comprising Hymenoptera, Coleoptera, Lepidoptera and Hemiptera (ML and BI trees, ML bootstrap values less than 80% and Bayesian posterior probabilities less than 0.95). In addition, termite ChREBP had an independent and accelerated evolution attested by an isolated clade with long branches.

**Figure 3.**
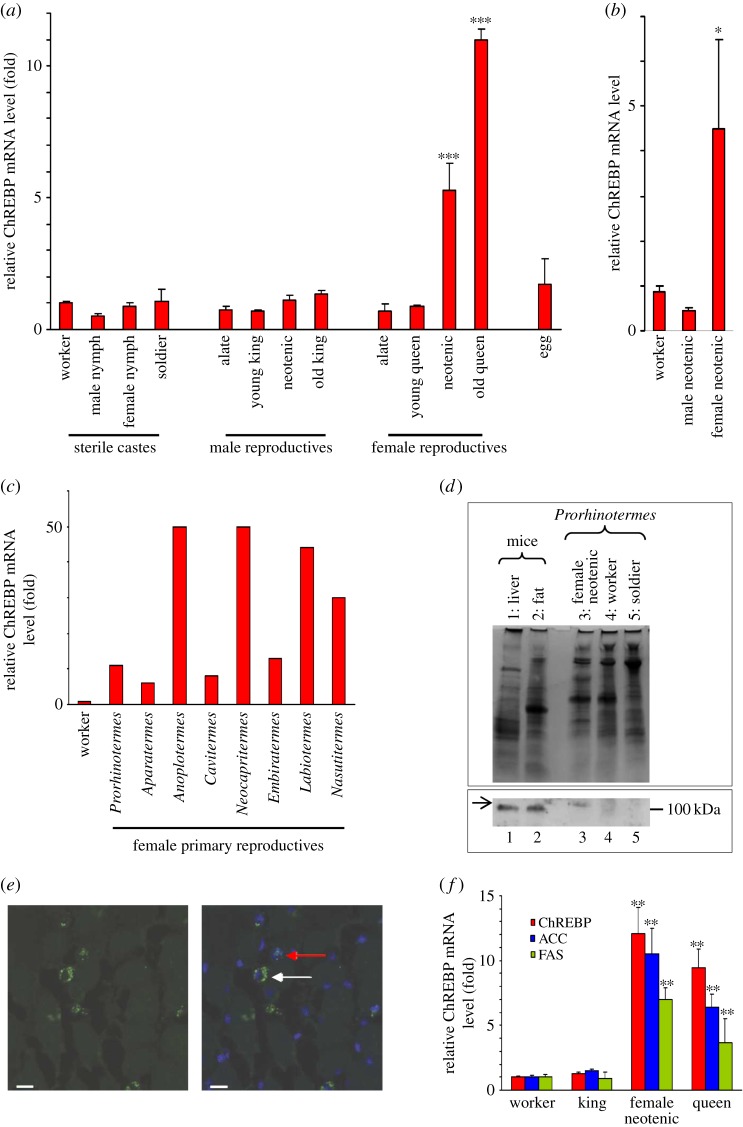
ChREBP and its effector lipogenic genes, ACC and FAS, are strongly upregulated in mature queens compared with kings and sterile individuals. (*a*) Expression of ChREBP mRNA in different castes and life stages of *Prorhinotermes canalifrons*. Total RNA was prepared and expression levels were determined by quantitative RT-PCR. Actin mRNA was used for normalization [29]. Reproductives were 4 months or 4 years old. Values are means ± s.e. (error bars) of at least 10 termites from each caste of four colonies reared in the laboratory. Significant differences (***p < 0.001) are indicated with asterisks. (*b*) Expression of ChREBP mRNA (qRT-PCR analysis) in different castes obtained during queenless experiments: in these conditions, some *P. canalifrons* workers are able to differentiate into male and female neotenics. Values are means ± s.e. (error bars) of three termites from three independent experiments. Significant differences from worker and male neotenics are indicated with asterisk (*p < 0.05). (*c*) Expression of ChREBP mRNA (qRT-PCR analysis) in female primary reproductives compared with workers in *Prorhinotermes* and in the Termitidae *Aparatermes*, *Anoplotermes*, *Cavitermes*, *Neocapritermes*, *Embiratermes*, *Labiotermes* and *Nasutitermes*. Columns represent the mean of collected workers (*n* = 10) and physogastric queens (*n* = 3). (*d*) Expression of ChREBP protein evaluated by western blot in *P. canalifrons* compared with mice tissues used as positive controls and efficiency of the antibodies (a representative blot is shown; upper panel: all proteins revealed by Coomassie staining; lower panel: western blot). Molecular marker (100 kDa) is provided. (1) Liver, (2) fat, (3) female reproductive (neotenic), (4) worker and (5) soldier. (*e*) Immunofluorescence image of the cellular ChREBP protein expression in a queen of *P. canalifrons*. Identification of nuclear (red arrow) and cytoplasmic (white arrow) ChREBP expression labelled using a commercial antibody against the human ChREBP peptide in green (left panel) and merged with nuclear DAPI stain in blue (right panel). Scale bars, 10 µm. (*f*) Expression of ChREBP, ACC and FAS mRNA (qRT-PCR analysis) in workers, male and female primary reproductives (king and queen, respectively), and female secondary reproductives (neotenic) of *P. canalifrons*. Values are means ± s.e. (error bars) of six termites from four independent colonies reared in the laboratory. Significant differences (**p < 0.01) from worker and male primary reproductive are indicated with asterisks. As values of workers are indicated as 1 arbitrarily for (*a*), (*c*) and (*f*), results are expressed according to the value of workers (fold).

Because of its high developmental plasticity, we investigated ChREBP expression in *P. canalifrons* in detail. Initially, we analysed ChREBP mRNA levels in all castes and developmental stages, including eggs, in four colonies. Quantitative real-time polymerase chain reaction (qRT-PCR) analysis using specific primers showed that ChREBP was upregulated in mature primary queens and in mature neotenic queens compared with young primary queens, alate females, male reproductives and sterile individuals (figure 3*a*). *Prorhinotermes canalifrons* workers are totipotent and can rapidly develop into male and female secondary reproductives (neotenics) when the primary queen or king disappears [35]. When the primary queen and king were experimentally removed, ChREBP mRNA levels increased during differentiation of sterile workers into mature female reproductives, but not into male reproductives (figure 3*b*). This suggested that ChREBP might play a role in the regulation of caste differentiation, specifically into the queen phenotype.

In Termitidae, and to a lesser extent in *P. canalifrons*, the queen exhibits a prodigious fecundity due to a gradual increase in the maturation of ovaries and continuous growth over time. This exceptional growth of an adult insect, known as physogastry, is unique to the termites [36]. We compared ChREBP expression in mature queens and sterile workers of eight different species, and for each species we found 10- to 50-fold increase in ChREBP expression in mature primary queens compared with their workers (figure 3*c*). Furthermore, the difference in ChREBP expression observed between young and mature queens represents a clear link between ChREBP and fertility, since young primary queens (4 months old) were only slightly physogastric, while mature primary queens (4 years old) had reached maximum physogastry (figure 3*a*). In addition, in all other termite species studied, the highest ChREBP expression was detected in the most physogastric and hence the most fertile queens based on the observed number of ovarioles (data not shown).

**Figure 4.**
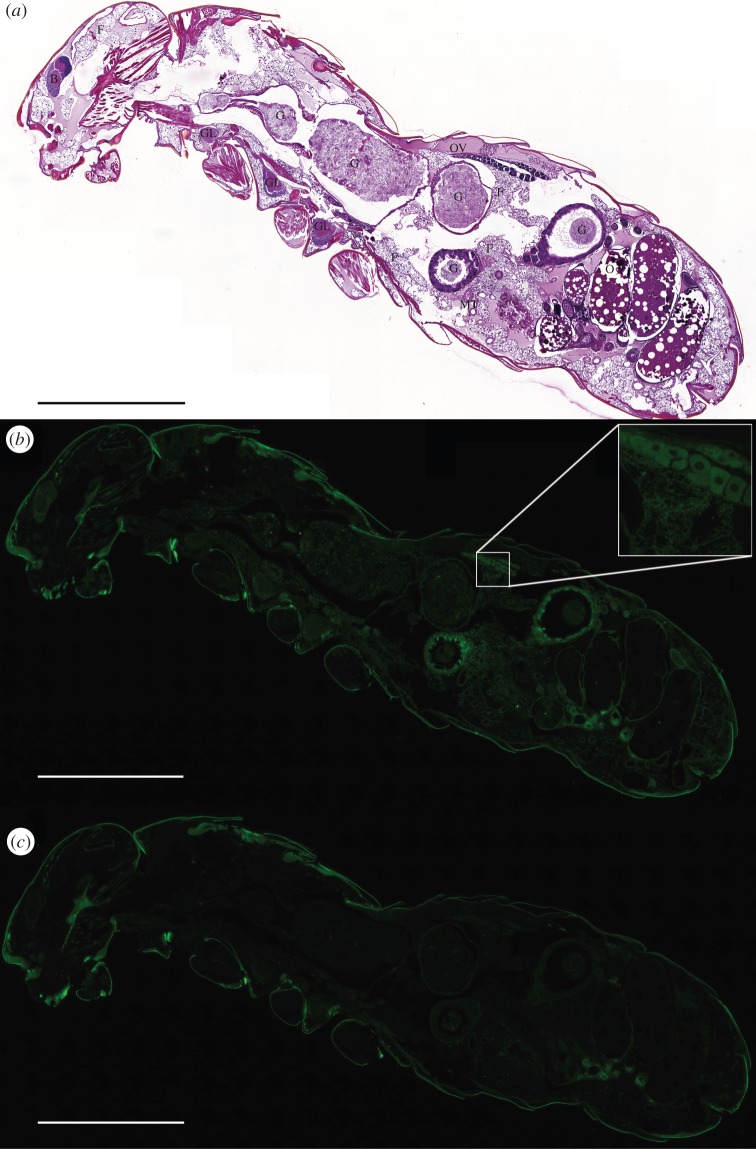
Lateral panoramic views of a physogastric primary queen of *Prorhinotermes canalifrons* showing ChREBP expression in metabolic tissues. (*a*) Hemalin–eosin staining: ovaries (O); ovarioles (Ov); fat tissue (F); brain (B); nervous ganglions (GL); gut (G); and Malpighian tubules (MT). (*b*) ChREBP immunostaining (in green) using a commercial antibody generated against the human ChREBP peptide. (*c*) Control of ChREBP immunostaining. This view confirms that ChREBP-dependent fluorescence is not a result of the non-specific binding of secondary antibodies. Examples of non-specific signals are found in the external cuticle, the hindgut cuticle and as dense material (possibly urates) in dorsal and ventral clusters of cells localized in the parietal fat body below the cuticle (green autofluorescence). Results are representative of three independent experiments. Scale bars, 1 mm.

By western blotting, we next quantified ChREBP protein levels in *P. canalifrons* using a commercial antibody generated against the human ChREBP peptide. As seen in the electronic supplementary material, figure S3, indeed this antibody showed a high specificity for the ChREBP termite protein. Consistent with the mRNA profile, the ChREBP protein was detectable in protein extracts from queens (figure 3*d*), and we also detected the protein at the cellular level using high-resolution panoramic colourimetric and immunohistological imaging (figure 4). ChREBP was observed in the cytoplasm and in nuclei (figure 3*e*) of different metabolic tissues, but was absent in muscles (figure 4). The absence of ChREBP in muscle tissue is consistent with the proposed hypothesis [16] that ChREBP represents the ancestral function of the ChREBP/Mondo A proteins. We detected ChREBP at high levels in germ cells, in oocytes at the beginning of vitellogenesis, and in somatic follicular cells (according to the nomenclature of Grandi [37,38]; figure 4). Furthermore, we detected ChREBP in certain cells of the villus of the crypt of the midgut and hindgut, and in Malpighian tubules (figure 4). The presence in the midgut may be particularly important, because this is where glucose is released during lignocellulose digestion [39]. Apart from the ovaries and the gut, ChREBP was detected in neurons of the protocerebrum and in ventral ganglions (figure 4). Finally, we observed expression of ChREBP in adipocytes of the fat body (figure 4), which is a functional counterpart to the mammalian adipose tissue and liver, which controls development, differentiation and metabolism by secretion of humoral factors [40]. In termites, the structure and the function of this complex tissue vary according to caste, sex and age [36,41]. Dramatic physiological modifications of adipocytes in fertile physogastric queens have previously been described at the structural level, when the imaginal adipocytes are transformed into royal adipocytes [36,42]. It should be noted that ChREBP mRNA levels determined in different tissues of *Drosophila* third instar larvae [16], and its enrichment reported in fat bodies of adult flies [43] were correlated with our protein cellular localization.

The localization of ChREBP in termite queen tissues, its function in sugar tolerance in the *Drosophila* fat body [16] and its role in the coordination to increase fat mass by regulating lipid synthesis [17] strongly suggest that ChREBP may be involved in the regulation of lipogenesis in mature termite queens. This hypothesis is consistent with the upregulation of mRNA levels of two genes coding for lipogenic enzymes, ACC and FAS (figure 3*f*), which are ChREBP target genes necessary for de novo synthesis of fatty acids [2,5,7,9].

### 2.2. Carbohydrate-responsive element-binding protein is regulated by a carbohydrate diet in termite queens

**Figure 5.**
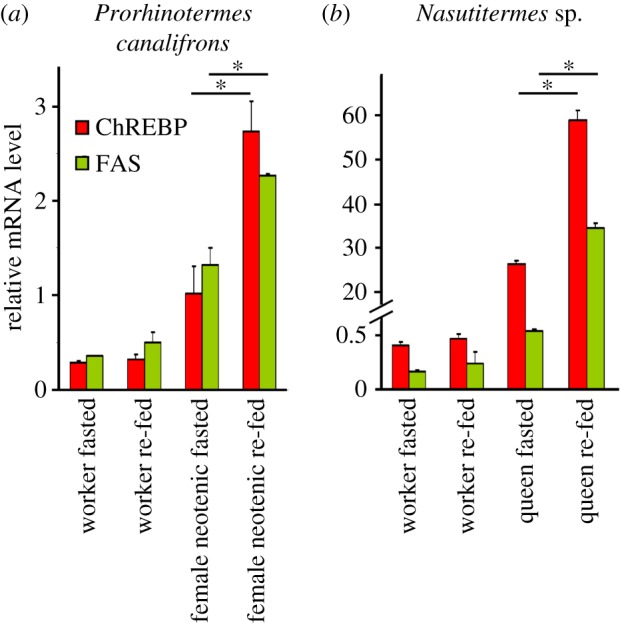
Dietary carbohydrates increase the abundance of ChREBP and FAS mRNA in queens. Expression of ChREBP mRNA and its FAS target gene (qRT-PCR analysis) in female neotenic reproductives compared with workers in *P. canalifrons* (*a*) and in primary queens compared with workers in *Nasutitermes* sp. (*b*)*.* Values are means ± s.e. (error bars) of 3–10 termites from three independent experiments. Significant differences from fasted are indicated with asterisk (*p < 0.05).

Based on the conserved structural organization of the functional domains on ChREBP, and the conserved regulation in mice [44,45] and *Drosophila* [46], where ChREBP mRNA levels increase when animals are fed high-carbohydrate diets, we decided to explore how ChREBP is regulated in relation to carbohydrate intake by *P. canalifrons* female neotenics and *Nasutitermes* sp. queens compared with workers. Indeed, we found a two to threefold increase in ChREBP mRNA levels in queens that were re-fed carbohydrates, in contrast to no changes in ChREBP mRNA levels in workers (figure 5); this increase in ChREBP expression in queens, in turn, leads to increased mRNA levels for genes encoding fatty-acid synthetic enzymes, such as FAS (figure 5).

### 2.3. Inhibition of fatty-acid synthesis in queens induces head-butting in workers, an early indicator of reproductive disinhibition

In order to test one of the hypothesized downstream effects of ChREBP, i.e. the regulation of synthesis of pheromones produced by mature queens to signal fertility, we combined a pharmacological approach and behavioural assays in *P. canalifrons*. In the absence of the fertility signal, e.g. in queenless colonies, secondary queens rapidly replace former queens. The lack of the queen pheromone signal and the resulting disinhibition of the reproductive potential in sterile immatures is evident from behavioural modification in prospective queens, as dominant workers likely to soon become the new reproductives increase head-butting [29,35,47]. To experimentally mimic the loss of the primary queen, we used 5-tetradecyloxy-2-furoic acid (TOFA), which inhibits fatty-acid synthesis of malonyl-CoA by ACC [48,49]. This modifies queen physiology and she is perceived by nest-mates as being absent. Butting behaviour among workers significantly increased when queens were fed TOFA (frequency of butting = 102 ± 10 (mean ± s.e.; *n* = 30)) compared with controls (21 ± 2; mean ± s.e.; *n* = 30; *p* < 0.05; Mann–Whitney *U*-test). This effect was absent when kings were fed TOFA (27 ± 3 versus controls 13 ± 2 (mean ± s.e.; *n* = 30; *p* = 0.81; Mann–Whitney *U*-test). Thus, activation of the ChREBP target gene ACC appears to be necessary for a queen to be perceived as a queen and to suppress the reproductive potential of sterile nest-mates.

## 3. Discussion

We have shown that the transcription factor ChREBP, an evolutionarily conserved glucose sensor that regulates gene expression to drive fatty-acid biosynthesis in mice and in the fruit fly, has a unique expression profile in termites, which are the oldest social insects. ChREBP is highly expressed in mature reproductive females of eight different termite species, compared with reproductive males and sterile worker and soldier individuals. ChREBP expression is increased in *P. canalifrons* workers, which become reproductive females, and when the ChREBP pathway is experimentally silenced dominant workers engage in head-butting behaviours as part of establishment of who takes over the reproduction. Collectively, these findings, in combination with the tissue localization of ChREBP expression in fat bodies and ovaries, strongly suggest that ChREBP is involved in fertility signalling and maintenance of reproductive dominance by the resident queen. We further document that dietary carbohydrates in the abdomen of mature female reproductives affect queen physiology, by inducing ChREBP expression and thus its lipogenic target genes.

Termites use cellulose as their main carbohydrate source, and glucose metabolism and its regulation are especially important as a signal of prodigious fecundity in reproductive females. Indeed, glucose acquired through adult feeding is especially important for females of longer lived species, who have to mature eggs throughout their adult life [50,51]. Recently, Foster *et al*. [52] demonstrated that adult-acquired carbohydrates are a major precursor (acetyl CoA) via incorporation into haemolymph trehalose and subsequent glycolysis for sex pheromone production in moths. In adults of *D. melanogaster*, lipid homeostasis influences pheromone production, since fat-body gene inactivation encoding lipid metabolic effectors, such as FAS, decreases the amount of pheromones [53,54]. In three eusocial insects, a conserved class of fatty-acid derived pheromones (saturated hydrocarbons) is overproduced by queens or fertile individuals and acts as sterility-inducing queen pheromones and as a signal of fecundity [28]. In termites, colony cohesion, expressed as queen reproductive monopoly, is maintained via the production of fatty-acid derived pheromones that operate as fertility signals to indicate the queen's presence and to prevent nest-mates from reproduction [29]. Using TOFA, an inhibitor of fatty-acid synthesis that functions by blocking the synthesis of malonyl-CoA by ACC, we demonstrated that the inhibition of fatty-acid synthesis of queens causes behavioural changes of workers with an increase of butting. Butting is associated with reproductive dominance, and workers that go on to replace the queen display more butting than workers that do not change caste [29,47]. This indicates that mature reproductive females produce pheromones derived from de novo fatty acids for suppression of worker head-butting behaviours, and we present substantive evidence supporting the fact that ChREBP is important in the production of these mature reproductive female signals because ACC is a target gene of ChREBP. This suggests that the activation of ChREBP, at least driven in part by carbohydrates, is necessary for the maintenance of reproductive dominance of the termite queen. This links the fertility network with the chemical communication pathway; however, the full pathway leading to the increase in ChREBP in mature female reproductives remains to be identified.

In all animals, digestion and absorption of carbohydrates in the diet induces profound hormonal changes, such as in the concentration of insulin. In most insects, including termites, two major hormones are essential to elicit major behavioural and physiological events. Juvenile hormone (JH) controls metamorphosis in immature insects [55–58] and maturation of reproduction in adult insects [59,60]. In adult females, JH levels are regulated in response to the intake of sugars [61] and JH interacts with insulin signalling [62,63]. The second hormone, ecdysone, is produced during adulthood primarily in the ovaries and accumulates at high levels in females, but not in males [64,65]. In flies, for example, ovary development results in the release of 20-hydroxyecdysone, which causes the production of cuticular hydrocarbon sex pheromone [66]. In the termite *Zootermopsis* (Archotermopsidae), JH and ecdysteroid titres were measured during ovarian maturation in young reproductive females following their release from inhibitory stimuli produced by mature queens. Four days after disinhibition, JH released by corpora allata and its titre in haemolymph decreased while ecdysteroid titre increased. Fully mature queens had the highest rate of JH production, the lowest ecdysteroid concentrations and the highest number of functional ovarioles [59]. Interestingly, in *Drosophila*, the sterol regulatory element-binding protein, a key regulator of lipid synthesis that functions in synergy with ChREBP to control lipogenic target genes [67], is activated by ecdysone to control oocyte lipid accumulation [68]. As ChREBP is expressed in somatic follicular cells and in the fat body (where JH induces the synthesis of the main precursor of egg yolk, i.e. vitellogenin), it is tempting to speculate that ChREBP could be regulated by JH and/or ecdysteroids in termites. This hormonal signalling may explain the caste and sex specificity in ChREBP gene expression.

In conclusion, ChREBP is a transcription factor upregulated in mature queens that, through a carbohydrate-rich diet, links nutritional status and endocrine control with the reproductive status of queens through the regulation of lipid metabolism. Our preliminary results also suggest that regulation of the lipid metabolism might be important in controlling termite phenotypic plasticity. The conservation of ChREBP in all termite species studied suggests that ChREBP has remained an important regulator over the course of millions of years of termite evolution.

## 4. Material and methods

### 4.1. Termites

In 2001, colonies of *P. canalifrons* (Rhinotermitidae) containing hundreds of individuals each were collected with pieces of wet wood on the Réunion Island in the Indian Ocean. Colonies were brought to the laboratory and reared at 28°C, 80% relative humidity and a 12 L : 12 D regime. Degraded birch wood was used as food. Cross-breeding of emerging alates was done in 2010 and in 2014, so that primary queens used in this study were 4 years or 4 months old, respectively. Colonies from Termitidae termite species (*Aparatermes* sp., *Anoplotermes* sp., *Cavitermes* sp*.*, *Neocapritermes taracua*, *Embiratermes* sp*.*, *Labiotermes labralis* and *Nasutitermes* sp.) were collected in French Guiana in March 2014. After removing their legs and antennae, individuals were put directly in RNAlater (Life Technologies) and maintained at −20°C.

### 4.2. Phylogenetic and amino acid alignment analyses

We obtained 45 amino acid sequences of ChREBP (also called ‘Mondo B’, ‘WBSCR14’, ‘MLXIPL’ or ‘MLX interacting protein-like’) for two vertebrates (human and mouse), one mollusc and 42 arthropods (wasps/sawflies, ants, bees, beetles, bugs, flies, moths, butterflies and a horseshoe crab) and amino acid sequences of Mondo A for two vertebrates (human and mouse) from the database at NCBI. Sequence searches were performed using ‘ChREBP alpha’, ‘WBSCR14’, ‘MLXIPL’, ‘MLX interacting protein-like’, ‘MondoB’, ‘Mondo’, ‘dMondo’, ‘dChREBP’ and ‘Mio’ keywords.

Amino acid sequences from the termites *Zootermopsis nevadensis* [30] and *Macrotermes natalensis* [31] and from the NCBI protein database were aligned using two different algorithms, Muscle and ClustalW2, with the Seaview software [69], and the alignment was checked manually. ChREBP is a multi-domain protein and some of its regions are highly conserved among the Mondo proteins, such as the Mondo conserved region (MCR) and the glucose-sensing module (GSM) that is the most important region in terms of glucose sensing and regulation [16,32]. Owing to its high conservation and structural role in the glucose response, only the GSM region was used in the phylogenetic study and alignment was trimmed according to the *Mus musculus* GSM region boundaries (accession no. NP067430) [16].

The best model of evolution was selected using ProtTest v. 3.3 software [70] and following the corrected Akaike Information Criterion. The best model of evolution identified by ProtTest was JTT + G (Jones Taylor Thornton model [71]). ML analyses were performed by PhyML [72] using an input tree generated by bioNJ, the JTT + G model of amino acid substitution and 1000 repetitions of bootstraps. BI analyses were performed by running two parallel analyses in MrBayes [73], each consisting of four Markov chains of 1 000 000 generations, each with a sampling frequency of one tree every one thousand generations and the JTT + G model of amino acid substitution. Convergence of the parameters was evaluated using Tracer v. 1.5.0 [74]. A consensus tree was then calculated after omitting the first 25% trees as burn-in.

### 4.3. Gene expression analysis

Total RNA was extracted from 3 mg of nitrogen-frozen crushed individual termites and purified using miRNeasy Micro kit (Qiagen) and RNAse-free DNAse according to the manufacturer's instructions (Qiagen). Reverse transcription was performed with 500 ng of total RNA using the iScript™ cDNA synthesis kit according to the manufacturer's protocol (Bio-Rad). Quantitative PCR was performed with 5 ng of reverse-transcribed total RNA, 0.5 µM of each primer (Eurogentec) in 1× Power SYBR Green PCR Master Mix (Life Technologies) using LightCycler StepOnePlus (Applied Biosystems). All samples were normalized to the threshold cycle value for actin mRNA, chosen as an invariant control [75]. Primer sequences will be provided on request. Amplifications of ChREBP, actin, ACC and FAS genes were checked by sequencing one unique amplified fragment per gene.

### 4.4. Western blotting and enhanced chemiluminescence detection

Termites, mice liver and adipocyte total extracts (40 mg each) were prepared using lysis buffer as described elsewhere [76]. Protein concentration was determined using the Bio-Rad Protein Assay. Proteins (50 µg) were subjected to SDS-PAGE analysis on 10% gels and transferred to nitrocellulose membranes. ChREBP proteins were detected with rabbit polyclonal antibody raised against a peptide mapping at the C terminus of ChREBP of human origin (1 : 5000 dilution of ab81958; 1 mg ml^−1^; Abcam). Liver and total fat protein extracts from glucose re-fed mice were used as positive controls of the experiments and efficiency of the antibodies (relative molecular weight 95 kDa). Blots were developed with ECL SuperSignal West Pico chemiluminescent reagents (Pierce).

To determine the specificity of binding of ChREBP antibody to ChREBP termite protein, *P. canalifrons* ChREBP cDNA was generated from queen poly(A) RNA and cloned in pT7CFE1-CHis vector (Thermoscientific). The protein was synthesized using *in vitro* TNT T7 Quick Coupled Transcription/Translation in Reticulocyte Lysate System (Promega) and analysed by western blotting. *In vitro* translated proteins (2 µl) were subjected to SDS-PAGE analysis on 12% gels and transferred to nitrocellulose membranes. *In vitro* synthesized ChREBP proteins were detected with rabbit polyclonal antibody raised against a peptide mapping at the C terminus of ChREBP of human origin (1 : 5000 dilution of ab81958; 1 mg ml^−1^; Abcam). We realized an immunizing peptide blocking experiment as recommended by Abcam. ChREBP antibody is neutralized, i.e. incubated with five to eight times excess blocking peptide (ab210715; 1 mg ml^−1^; Abcam) that corresponds to the epitope recognized by the antibody, to antibody by weight during hybridization overnight at 4°C. Blots were developed with Clarity™ Western ECL Blotting Substrate on ChemiDoc MP (BioRad).

### 4.5. Immunohistochemistry

*Prorhinotermes canalifrons and Nasutitermes* sp*.* mature queens were fixed for 24 h in 4% paraformaldehyde and embedded in paraffin. Serial sections (4 µm) were immunostained for ChREBP overnight at 4°C using rabbit polyclonal anti-ChREBP antibody (1 : 500 dilution of ab81958; 1 mg ml^−1^; Abcam) followed by a fluorescein isothiocyanate-conjugated goat anti-rabbit secondary antibody (1 : 300; eBioscience). Sections were mounted using Vectashield mounting medium with DAPI (4′-6-diamidino-2-phenylindole; Vector Laboratories), scanned using the Pannoramic Lamina multilabel slide scanner (Perkin Elmer) and observed with Pannoramic viewer and ImageJ. To determine the specificity of binding of ChREBP antibody to ChREBP termite protein, we also realized immunizing peptide blocking experiments, as recommended by Abcam, on sections of *P. canalifrons* and *Nasutitermes* sp*.* mature queens. Before proceeding with the staining protocol, ChREBP antibody (1 : 500 dilution of ab81958; 1 mg ml^−1^; Abcam) is neutralized, i.e. incubated with five times excess blocking peptide (ab210715; 1 mg ml^−1^; Abcam) that corresponds to the epitope recognized by the antibody, to antibody by weight overnight at 4°C.

### 4.6. Feeding experiments

Physogastric queens, kings and 100 workers of *Nasutitermes* sp. and physogastric neotenic female reproductives, neotenic male reproductives and 30 workers of *P. canalifrons* from six colonies per species were placed in Petri dishes on humidified sand, fasted for 24 h, and then fed for 24 h (*Nasutitermes* sp.) and 30 h (*P. canalifrons*) with a piece of wood impregnated with 10% glucose. Individuals were weighed before to start the experiment and after the diet protocol to observe losses and/or gains of weight. Nitrogen-frozen crushed female neotenic and queen abdomen and worker extracts were prepared and analysed for actin, ChREBP and FAS mRNA levels.

### 4.7. Effect of 5-tetradecyloxy-2-furoic acid on behaviour

Physogastric secondary queens and kings of *P. canalifrons* from three colonies were isolated in Eppendorf tubes pierced with numerous small openings and filled with humidified sand. Each tube was placed in a Petri dish (ø = 5 cm) containing 30 workers from the same colony. This set-up allowed antennation among workers and the diffusion of volatiles emitted by the reproductives into the Petri dish. Reproductives were fed for 3 days with 0.5 cm^2^ square paper (Whatman 3MM) loaded with 10 µl of TOFA (99% purity; Santa Cruz Biotechnology) suspended in ethanol at a concentration of 3 mg ml^−1^ or with 10 µl of ethanol for control experiments. It is known that TOFA is converted into 5-tetradecyloxy-2-furoyl-CoA exerting an allosteric inhibition on ACC that prevents fatty-acid synthesis in adipocytes and hepatocytes [48,49]. Workers in the Petri dishes had access to non-treated Whatman 3MM paper only. Nile blue dye allowed us to validate the feeding efficiency as previously reported [75]. Queens and kings started to feed approximately 15 h after the introduction of the square paper, which was weighed 1 and 3 days after introductions. Prior to these experiments, we verified the viability of reproductives in response to TOFA concentrations; no mortality was observed. Three days after the introduction of the paper, we measured the frequency of head-butting interactions among workers (as defined in [47]) in each dish by recording for 30 min with a Sony DCR-SR90 camera.

A control experiment, adapted from Penick *et al.* [47], was conducted using three colonies to obtain a reference of butting behaviour in this termite species in queenright and queenless conditions. Briefly, we allowed one queen with 60 workers to acclimate for three days to the new nest conditions (Petri dish with Whatman 3MM paper). Then, 30 workers and the queen were placed in one dish while the other 30 workers were placed in another dish for 20 h without the queen. Subsequently, head-butting events among workers were recorded for 30 min. Removing queens from colonies resulted in a significant increase in butting behaviour among workers in queenless (40 ± 10; mean ± s.e.; *n* = 30) compared with queenright (10 ± 3; mean ± s.e.; *n* = 30; *p* < 0.04, Mann–Whitney *U*-test) conditions, similar to what has been observed in *Z. nevadensis* [47] and *Cryptotermes secundus* [77].

To determine if inhibition of fatty-acid synthesis by TOFA caused a change in other semiochemical profiles of the workers, we recorded trail-following pheromone bioassays as described previously [78]. We observed no significant differences between worker behaviour when queens were fed with TOFA or ethanol or when kings were fed with TOFA or ethanol (electronic supplementary material, table S1). In the light of the phenotype observed in ACC gene knockout *Drosophila* [53], we dissected several reproductives and used binocular scopes to determine cuticular defects, but none were observed.

### 4.8. Developmental experiments

Nine groups of 20 old workers of *P. canalifrons* were collected from three colonies and kept in three Petri dishes containing humidified sand and supplied with pieces of birch wood. The groups were monitored daily. Three weeks of absence of reproductives induced development of new reproductives, male and female neotenics, in all nine experimental groups. Eggs were observed in all groups as well. Total RNA was extracted and purified from individual termites and ChREBP expression was quantified as reported above.

### 4.9. Statistics

Quantitative results are expressed as means ± s.e. The comparison of different groups was carried out using unpaired two-tailed Student's *t*-test. For statistical analyses with non-normal distributions or unequal variances, analyses were carried out using Mann–Whitney *U*-test. Differences were considered statistically significant at *p* < 0.05.

## Supplementary Material

Supplementary Figures and Table
