# Correction to ‘The role of the glucose-sensing transcription factor carbohydrate-responsive element-binding protein pathway in termite queen fertility’

**DOI:** 10.1098/rsob.160237

**Published:** 2016-10-12

**Authors:** David Sillam-Dussès, Robert Hanus, Michael Poulsen, Virginie Roy, Maryline Favier, Mireille Vasseur-Cognet

*Open Biol.* **6**, 160080. (2016; Published online 18 May 2016) (doi:10.1098/rsob.160080)

The correct affiliation for this author is as follows:

Mireille Vasseur-Cognet^2,3,4,8^

^1^Université Paris 13, Laboratoire d'Ethologie Expérimentale et Comparée, EA4443, 93430 Villetaneuse, France

^2^UMR IRD 242, UPEC, CNRS 7618, UPMC 113, INRA 1392, PARIS 7 113, Institut d'Ecologie et des Sciences de l'Environnement de Paris, 93140 Bondy, France

^3^Sorbonne Paris Cité, Paris, France

^4^Sorbonne Universités, Paris, France

^5^Institute of Organic Chemistry and Biochemistry, Academy of Sciences of the Czech Republic, 16610 Prague, Czech Republic

^6^Centre for Social Evolution, Section for Ecology and Evolution, Department of Biology, University of Copenhagen, 2100 Copenhagen East, Denmark

^7^Institut National de la Santé et de la Recherche Médicale, Unité 1016, Institut Cochin, 75014 Paris, France

^8^Institut National de la Santé et de la Recherche Médicale, Paris, France

