# Supplementary material for: The role of the glucose-sensing transcription factor carbohydrate-responsive element-binding protein pathway in termite queen fertility
Source: Open Biol. 2016 May 18;6(5):160080. doi: 10.1098/rsob.160080 (PMC4892437; doi:10.1098/rsob.160080)
Supplement: Supplementary Figures and Table [file rsob160080supp1.pdf]

Electronic Supplementary Information for

The role of the glucose-sensing transcription factor ChREBP pathway in termite queen fertility.

David Sillam-Dussès, Robert Hanus, Michael Poulsen, Virginie Roy, Maryline Favier and Mireille Vasseur-Cognet*

* Corresponding author email: mireille.vasseur@inserm.fr

Figure S1: The alignment of ChREBP ortholog amino acid sequences shows an evolutionarily conserved ChREBP protein sequence between vertebrates and invertebrates. Amino acid sequences of ChREBP from (A) *Homo sapiens* (NP_116569), (B) the mouse *Mus musculus* (NP_067430), (C) the fruit fly *Drosophila melanogaster* (NP724326), (D) the honeybee *Apis mellifera* (XP_394429), (E) the basal termite *Zootermopsis nevadensis* (provided from [S1]), (F) the Termitidae *Macrotermes natalensis* (provided from [S2]). The first sequence is the *Homo sapiens* reference; in the other sequences, the residue identical to the first sequence residue at the same position is represented by a dot. A residue that is highly conserved (consensus value = 90%) appears in red and as an uppercase letter in the consensus line. A residue that is weakly conserved (consensus value = 50%) appears in blue and as a lowercase letter in the consensus line. Other residues appear in black. A position with no conserved residue is represented by a dot in the consensus line. Near the N terminus, ChREBP alpha isoform contains the GSM domain, two nuclear export signals and a nuclear localization signal. In the central part, several proline-rich domains are situated, implicated in protein-protein interactions. In the C terminal region, a conserved nuclear receptor box (LxQLLT motif) and a transcriptional activity region includes a basic helix-loop-helix/leucine zipper like DNA binding domain (bHLH/LZ) [S3].

Figure S2: Bayesian inference analyses. Fifty percent majority rule consensus tree obtained from the Bayesian inference analyses of ChREBP amino acid sequences (GSM region, 331 aa). Bayesian posterior probabilities > 0.95 and ML bootstrap values > 80% are plotted on the nodes. Social species are indicated in bold. Tree is rooted on vertebrate Mondo A and Mondo B sequences.

Very similar topologies were obtained with ChREBP entire sequence (data not shown). Upper left corner: insect phylogeny adapted from [S4] and [S5].

Figure S3: The commercial antibody generated against the human ChREBP peptide specifically recognizes the termite ChREBP protein. There is a 60% of amino acid sequence identity between human and termite ChREBP in the C terminal region used to generate the ChREBP antibody. To address the specificity of the ChREBP antibody to the termite ChREBP protein, we realized (a) *in vitro* translated termite ChREBP protein in peptide competition immunoblotting experiments and (b) in peptide competition immunohistochemistry experiment. (a): Upper panel is a picture of loaded samples on a TGX Stain-Free™ FastCast™ 12% acrylamide gel (BIORAD) and lower panel is a representative Western blot with 1- *in vitro* translated ChREBP termite plus ChREBP antibody, 2- *in vitro* translated ChREBP termite plus ChREBP antibody plus with five times excess blocking peptide, and 3- *in vitro* translated ChREBP termite plus ChREBP antibody plus with eight times excess blocking peptide. (b): ChREBP immunostaining (in green) and nuclei were specifically stained using DAPI (blue). Signal (in figure a, in lines 2 and 3 compared to 1, and in figure b) decrease with ChREBP antibody plus an excess of blocking peptide. Non-specific signals are found in the external cuticle, and in the parietal fat body below the cuticle (green autofluorescence) as dense material (possibly urates) in dorsal and ventral clusters of cells localized. Scales= 200 μm.

Table S1: Trail-following bioassays performed with *P. canalifrons* workers. The distance traveled by workers on artificial trails made of worker sternal gland extracts (10^{-1} gland equivalent/cm) is measured (Mean ± SD; n=10).

Supplemental references

S1. Terrapon N *et al*. 2014 Molecular traces of alternative social organization in a termite genome. *Nat. Commun.* **5**, 3636. (doi:10.1038/ncomms4636)

S2. Poulsen M *et al*. 2014 Complementary symbiont contributions to plant decomposition in a fungus-farming termite. *Proc. Natl Acad. Sci.* **111**, 14500 – 14505. (doi:10.1073/pnas.1319718111)

S3. Filhoulaud G, Guilmeau S, Dentin R, Girard J, Postic C. 2013 Novel insights into ChREBP regulation and function. *Trends Endocrin. Met.* **24**, 257-268. (doi:10.1016/j.tem.2013.01.003)

S4. Mao M, Gibson T, Dowton M. 2015 Higher-level phylogeny of the Hymenoptera inferred from mitochondrial genomes. *Mol. Phylogenet. Evol.* **84**, 34 – 43. (doi:10.1016/j.ympev.2014.12.009)

S5. Misof *et al*. 2014 Phylogenomics resolves the timing and pattern of insect evolution. *Science* **346**, 763 – 767. (doi:10.1126/science.1257570)
